# Altered sulcogyral patterns of orbitofrontal cortex in a large cohort of patients with schizophrenia

**DOI:** 10.1038/s41537-016-0008-y

**Published:** 2017-01-12

**Authors:** Shuichi Isomura, Ryota Hashimoto, Motoaki Nakamura, Yoji Hirano, Fumio Yamashita, Shin Jimbo, Hidenaga Yamamori, Michiko Fujimoto, Yuka Yasuda, Ryan P. Mears, Toshiaki Onitsuka

**Affiliations:** 1 Department of Neuropsychiatry, Graduate School of Medical Sciences, Kyushu University, 3-1-1, Maidashi, Higashiku, Fukuoka, Japan; 2 Molecular Research Center for Children’s Mental Development, United Graduate School of Child Development, Osaka University, Osaka, Japan; 3 Department of Psychiatry, Osaka University Graduate School of Medicine, Osaka, Japan; 4 Kanagawa Psychiatric Center, Kanagawa, Japan; 5 Division of Ultra-high Field MRI, Institute for Biomedical Sciences, Iwate Medical University, Iwate, Japan; 6 Department of Psychology, University of Florida, Gainesville, FL USA

## Abstract

Abnormalities in prenatal brain development contribute to schizophrenia vulnerability. Orbitofrontal cortex sulcogyral patterns are largely determined during prenatal development, and four types of orbitofrontal cortex sulcogyral patterns have been classified in humans. Altered orbitofrontal cortex patterns have been reported in individuals with schizophrenia using magnetic resonance imaging; however, sample sizes of previous studies were small–medium effects for detection, and gender manifestation for orbitofrontal cortex sulcogyral patterns is unclear. The present study investigated orbitofrontal cortex patterns of 155 patients with schizophrenia and 375 healthy subjects. The orbitofrontal cortex sulcogyral pattern distributions of schizophrenia were significantly different compared with healthy subjects in the left hemisphere (*χ* ^2^ = 14.55, *p* = 0.002). In female schizophrenia, post-hoc analyses revealed significantly decreased Type I expression (*χ* ^2^ = 6.76, *p* = 0.009) and increased Type II expression (*χ* ^2^ = 11.56, *p* = 0.001) in the left hemisphere. The present study suggested that female schizophrenia showed altered orbitofrontal cortex patterns in the left hemisphere, which may be related to neurodevelopmental abnormality.

## Introduction

Abnormalities in prenatal brain development contribute to schizophrenia (SZ) vulnerability. Accumulating evidence suggests that the orbitofrontal (OFC) sulcogyral pattern could be used as morphometric trait marker of psychosis.^1–6^ The gross anatomy of the OFC varies widely between individuals; OFC sulcogyral patterns are classified into four types, Types I–IV.^1–6^ Since OFC sulcogyral patterns are largely determined during prenatal development and independent of longitudinal changes after birth,^7^ it is possible to examine these patterns to gain insight into abnormal perinatal brain development in SZ. Magnetic resonance imaging (MRI) studies of OFC have consistently demonstrated the most frequent pattern, Type I, was decreased in SZ. However, sample sizes of previous studies were small-medium effects for detection. Moreover, as shown in supplementary information (Supplementary SI-1), samples of the previous studies are predominantly male subjects and gender manifestation for OFC sulcogyral patterns is unclear. Therefore, this study assessed OFC sulcogyral pattern in a larger cohort of subjects (*N* = 530), and also investigated gender effects.

## Results

**Fig. 1:**
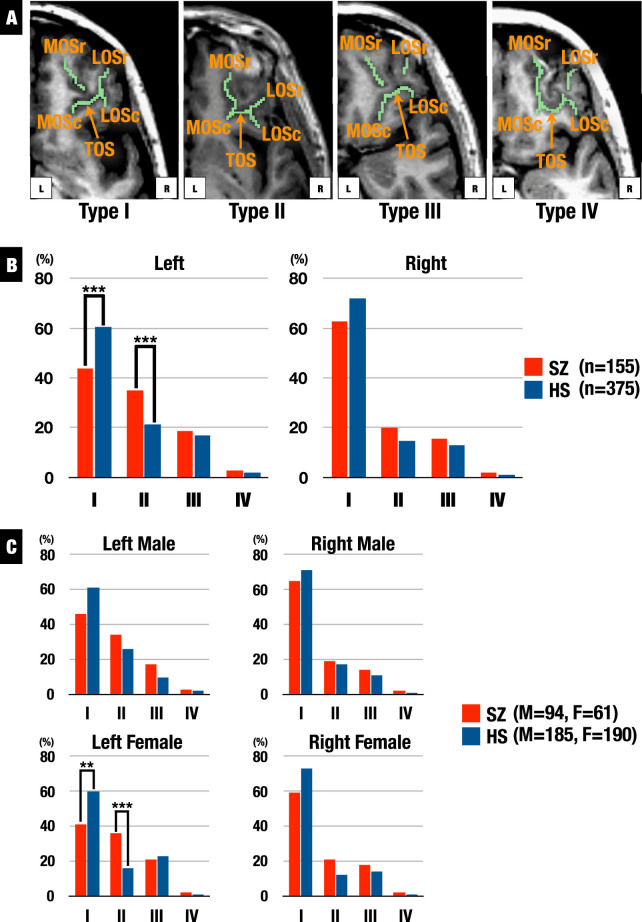
**a** Classification of the orbitofrontal cortex sulcogyral pattern with MRI. Type I: rostral and caudal portions of the LOS were connected while the MOS were clearly interrupted between rostral and caudal portions of MOS, Type II: rostral and caudal portions of both LOS and MOS were connected, Type III: rostral and caudal portions of both LOS and MOS were interrupted, Type IV: rostral and caudal portions of the MOS were connected while the LOS were interrupted. (*LOS* lateral orbital sulcus, *MOS* medial orbital sulcus, *TOS* transverse orbital sulcus, -*r* rostral, -*c* caudal). **b** Distributions of the orbitofrontal cortex sulcogyral pattern in patients with schizophrenia (SZ) and healthy subjects (HS) in each hemisphere. Patients showed decreased Type I expression (HS > SZ: *χ* ^2^ = 12.34, *p* < 0.001) and increased Type II expression (HS < SZ: *χ* ^*2*^ = 11.07, *p* < 0.001) in the left hemisphere. *** *p* < 0.001. **c** Distributions of the orbitofrontal cortex sulcogyral pattern for each gender in patients with SZ and HS in each hemisphere. Female patients showed decreased Type I expression (HS > SZ: *χ* ^*2*^ = 6.76, *p* = 0.009) and increased Type II expression (HS < SZ: *χ* ^*2*^ = 11.56, *p* < 0.001) in the left hemisphere. ** *p* < 0.01, *** *p* < 0.001

The demographic data of the subjects for each gender are shown in Table 1. SZ OFC distribution patterns significantly differed from healthy subjects (HS) in the left (*χ* ^2^ = 14.55, *p* = 0.002), but not the right (*χ* ^2^ = 5.38, *p* = 0.15) hemisphere (Fig. 1b).

**Table 1:** Demographic and clinical characteristics of subjects for each gender

	Healthy Subjects	Patients with Schizophrenia	*t* or *χ*2	*p*
Male subjects	*N* = 185	*N* = 94		
Age (years)	36.3 ± 13.0	34.3 ± 10.9	1.9	0.22
Education (years)	15.4 ± 2.3	14.4 ± 2.6	3.4	0.01
IQ	111.8 ± 12.1 (*N* = 181)	88.6 ± 18.1 (*N* = 81)	12.0	<0.001
Handedness (right/left)	171/14	88/6	0.1	0.81
Onset (years)		23.7 ± 8.3		
Duration of illness (years)		10.6 ± 8.8		
PANSS positive		14.5 ± 5.1 (*N* = 92)		
PANSS negative		18.7 ± 5.6 (*N* = 92)		
Chlorpromazine equivalent dose (mg)		580.8 ± 554.2		
Female subjects	*N* = 190	*N* = 61		
Age (years)	36.8 ± 12.6	37.9 ± 13.0	−0.6	0.59
Education (years)	14.6 ± 2.2	13.7 ± 2.2	2.6	0.01
IQ	108.2 ± 12.2 (*N* = 187)	83.3 ± 16.3 (*N* = 45)	11.5	<0.001
Handedness (right/left)	182/8	59/2	0.1	1.00
Onset (years)		24.7 ± 10.2		
Duration of illness (years)		13.1 ± 9.9		
PANSS positive		14.3 ± 4.6 (*N* = 58)		
PANSS negative		16.3 ± 5.8 (*N* = 58)		
Chlorpromazine equivalent dose (mg)		637.2 ± 604.5

To evaluate gender effects, *χ* ^2^ tests were performed for each gender. In females, patients exhibited altered OFC patterns in left (*χ* ^2^ = 12.38, *p* = 0.006) but not in right (*χ* ^2^ = 5.04, *p* = 0.17) compared with HS. In female SZ, post-hoc analyses revealed significantly decreased Type I expression (*χ* ^2^ = 6.76, *p* = 0.009) and increased Type II expression (*χ* ^2^ = 11.56, *p* = 0.001) in left (Fig. 1c). In male subjects, OFC patterns of SZ were not significantly different from HC in both hemispheres (*χ* ^2^ = 6.38, *p* = 0.1 for left, *χ* ^2^ = 2.21, *p* = 0.53 for right).

Since Nakamura *et al*.^1^ reported that SZ patients with type III expression evinced poorer cognitive function compared to patients without type III expression, we have assessed IQ difference between female subjects with and without type III expressions in the left hemisphere. Type IV was excluded in the analysis, because pathophysiological significances are unknown. Female patients with type III showed significantly lower IQ (74.7 ± 10.8) compared to patients without type III (85.6 ± 17.1) (*t*[55] = −2.4, *p* = 0.03).

## Discussion

In the present large-sample study, OFC sulcogyral patterns were significantly altered in SZ compared to HS, and this finding was partially consistent with previous findings.^1–3, 5^ Comparable with previous studies, right hemisphere Type I OFC pattern diminished significantly in SZ.^1, 3, 5^ In contrast to previous studies,^1–3, 5^ we found altered OFC sulcogyral patterns in the left hemisphere in female SZ. There was significantly lower prevalence of Type I and significantly higher prevalence of Type II patterns in the left hemisphere. The recent MRI study reported abnormal left hemispheric OFC patterns in adolescents born extremely preterm and/or at an extremely low birth weight.^8^ The sulcogyral pattern of the brain is formed during neurodevelopment, and it may be possible that our female SZ sample presented altered OFC patterns in the left hemisphere. Previous studies have reported that development of the right hemisphere occurs earlier than that of the left,^9^ and the authors Ref. 8 speculated that a prolonged period of vulnerability for the left hemisphere is related to the increased chance of abnormal development.

Clinically, the present study showed that subjects with type III showed significantly lower IQ in female SZ. Type III expression may be a part of a neurodevelopmental alteration; however, further study will be needed in a larger female SZ subjects.

Precise reasons for discrepancies between the current results and previous findings are unclear. However, frequencies of Types II, III, and IV expressions are lower compared to Type I, and hence whether statistical significance is found may depend heavily on the subject group evaluated. Although the present investigation is the first to detect OFC pattern effects in the left hemisphere in SZ, limited sample size prevented prior investigations from sufficiently exploring small to medium effect sizes (see Supplementary SI-2, SI-3).

In conclusion, the present study revealed the diminished Type I and the increased Type II OFC sulcogyral patterns in the left hemisphere in female SZ.

## Methods

MRI images of 158 SZ and 378 HS were investigated in this study. Three patients and three HS withdrew their consents for this study, so they were excluded. The remaining subjects were the same as those in our most recently published MRI study,^8^ and the subject recruitment, inclusion criteria and diagnostic evaluations have been described in the study.^10^ This study was approved by the Research Ethical Committee of Osaka University.

The MRI series was performed on a 1.5T Magnetom Symphony (Siemens). The protocol followed that of the previous publication.^10^ For consistent identification of the sulcogyral pattern, images were realigned using the line between the anterior and posterior commissures and the mid-sagittal plane to correct any head tilt, and resampled into isotropic voxels (0.9375 mm^3^). We used the OFC pattern classification by the method of Nakamura *et al*.,^1^ and Type IV was undifferentiated type. Classification methods for the OFC sulcogyral patterns have been described in detail elsewhere^1^ (see Fig. 1a).

We used medical image analysis software packages (3D slicer, www.slicer.org), and the sulcogyral pattern classification in each hemisphere of the 530 subjects was done by S.I., who was blinded to the diagnoses. Intraclass correlation coefficients were computed for the sulcal patterns by three independent raters, who were also blinded to the diagnoses. Thirty cases were selected randomly for interrater reliability. The intraclass correlation coefficients were 0.95 for left and 0.92 for right.

To evaluate group differences in sulcogyral pattern distribution, *χ* ^2^ tests were applied to each hemisphere. For all statistical tests, *α* was set to 0.05.

## Electronic supplementary material

Supplementary Information
