# Supplementary material for: Altered sulcogyral patterns of orbitofrontal cortex in a large cohort of patients with schizophrenia
Source: NPJ Schizophr. 2017 Jan 12;3:3. doi: 10.1038/s41537-016-0008-y (PMC5441528; doi:10.1038/s41537-016-0008-y)
Supplement: Supplementary file 1 — Supplementary Information [file 41537_2016_8_MOESM1_ESM.docx]

**Supplementary Information (SI)**

**SI-1 Table**

**SI-2 Figure**

SI-2 Figure. A shaded line plot of statistical power for Chi-Squared (*df* = 2) illustrates the required sample sizes to detect effects at each of four power levels, .5, .65, .8, and .95. For further comparison of power between the main findings from several investigations, points corresponding to effect size (*w*) and sample size (N) accompany the shaded lines of the statistical power model. Point shapes correspond to right (square) and left (circle) hemispheres and male (hatched square or circle for male right or left) and female (unenclosed plus or asterisk for female right or left). Citation number accompanies each point from other investigations. Statistical power greater than 0.8 is considered to be sufficiently sensitive to be reliable for all effect sizes, and in this figure only a few results are above the .8 power curve. In the present study, the left hemisphere result for HC vs. SZ and the female left hemisphere result for HC vs. SZ have greater than .8 power for small to medium effect sizes. For the right hemisphere results from two studies^1,6^, results have greater than .8 power for very large effect sizes.

^3^ Bartholomeusz et al. 2013, ^1^ Nakamura et al. 2007, ^6^ Uehara-Aoyama et al. 2011, ^&^ Chiavaras et al. 2000

**SI-3 Table**

|  |  |  | |  |  | |  | |  |  |
| --- | --- | --- | --- | --- | --- | --- | --- | --- | --- | --- |
| Results for OFC type | Condition | *χ^2^* | *w* | *1-β* | | *df* | | N | |  |
| Overall: SZ vs. HC ^#^ | Right | 5.38 | 0.1 | 0.47 | | 3 | | 536 | |  |
|  | Left | 14.55* | 0.16 | 0.91 | | 3 | | 536 | |  |
| Male SZ vs. HC ^#^ | Right | 6.38 | 0.15 | 0.54 | | 3 | | 279 | |  |
|  | Left | 2.21 | 0.09 | 0.21 | | 3 | | 279 | |  |
| Female SZ vs. HC ^#^ | Right | 5.04 | 0.14 | 0.44 | | 3 | | 251 | |  |
|  | Left | 12.38* | 0.22 | 0.85 | | 3 | | 251 | |  |
| Overall: FEP vs. HC ^3^ | Right | 8.53* | 0.22 | 0.75 | | 2 | | 169 | |  |
|  | Left | 1.81 | 0.1 | 0.21 | | 2 | | 169 | |  |
| Overall: SZ vs. HC ^1^ | Right | 13.67* | 0.52 | 0.96 | | 2 | | 50 | |  |
|  | Left | 2.23 | 0.21 | 0.32 | | 2 | | 50 | |  |
| Overall: SZ vs. HC ^6^ | Right | 0.98 | 0.14 | 0.13 | | 2 | | 47 | |  |
|  | Left | 0.47 | 0.1 | 0.09 | | 2 | | 47 | |  |
| Overall: HC only ^&^ | Right | 23.08* | 0.68 | 0.99 | | 2 | | 50 | |  |
|  | Left | 6.76 | 0.37 | 0.64 | | 2 | | 50 | |  |
|  |  |  |  |  | |  | |  | |  |

^#^ Present results, ^3^ Bartholomeusz et al. 2013, ^1^ Nakamura et al. 2007, ^6^ Uehara-Aoyama et al. 2011,

^&^ Chiavaras et al. 2000. SZ: Schizophrenia, FEP: First Episode Psychosis, HC: Healthy Controls, *p* <.05*

**SI-3 Table**

Comparison of Effect sizes (*w*) and Power (*1 - β*) for χ^2^ analyses of OFC type

Prior studies achieved sufficient power (*1 - β* ≥ 0.8) to detect medium to large effect sizes (*w* > 0.3) but not medium to small effects (*w* < 0.3). The type II error rate (β) is defined as the likelihood of accepting the null hypothesis when it is false, and power (*1 - β*) is defined as the likelihood of correct rejection for a false null hypothesis. Whereas Chi-squared (*χ^2^*) value, degrees of freedom (*df*), and alpha (*α*) were defined from the main results of this investigation and several others, effect sizes (*w*) were calculated from chi-square and sample size (N). $w=\sqrt{\frac{\chi^{2}}{N}}$ . Post-hoc power was calculated in G*Power ^a^. The primary results of the investigation were sufficiently powered. In the present study, gender differences moderate the left hemisphere OFC pattern in SZ, an effect that was not previously found.

**References**

a. Faul F, Erdfelder E, Lang AG, & Buchner A. G*Power 3: A flexible statistical power analysis program for the social, behavioral, and biomedical sciences. Behavior Research Methods. 2007; 39: 175-191.
